# Epidermoid Cyst in an Intrapancreatic Accessory Spleen Complicating Clinical Decision-Making: A Case Report With Characteristic Imaging Findings

**DOI:** 10.7759/cureus.69957

**Published:** 2024-09-22

**Authors:** Rin Tsujimoto, Ryo Kurokawa, Amane Yamamoto, Yoshikuni Kawaguchi, Mari Miyashita, Kiyoshi Hasegawa, Osamu Abe

**Affiliations:** 1 Radiology, The University of Tokyo, Tokyo, JPN; 2 Pathology, The University of Tokyo, Tokyo, JPN; 3 Hepato-Biliary-Pancreatic Surgery, The University of Tokyo, Tokyo, JPN

**Keywords:** ct, epidermoid cyst, intrapancreatic accessory spleen, mri, superparamagnetic iron oxide

## Abstract

Epidermoid cyst in intrapancreatic accessory spleen (ECIPAS) is a rare benign condition that occasionally mimic malignant pancreatic neoplasms. We present a case of ECIPAS in a 53-year-old asymptomatic male, initially discovered incidentally during imaging for a suspected hepatic hemangioma. The lesion, located in the pancreatic tail, demonstrated characteristic imaging features on contrast-enhanced computed tomography and superparamagnetic iron oxide (SPIO)-enhanced magnetic resonance imaging (MRI), including a cystic component with peripheral solid tissue exhibiting splenic enhancement patterns. Despite these typical ECIPAS findings, the lesion increased in size from 38 × 33 mm to 50 × 45 mm over 12 months, accompanied by a significant rise in serum carbohydrate antigen 19-9 (CA19-9) from 21 to 330 U/mL. This clinical progression raised concerns about potential malignancy, leading to a robot-assisted spleen-preserving distal pancreatectomy. Histopathological examination confirmed the diagnosis of ECIPAS. Postoperatively, the patient's serum CA19-9 levels normalized. This case highlights that ECIPAS can complicate clinical decision-making through size increase and CA19-9 elevation, complicating preoperative diagnosis. However, careful analysis of imaging characteristics, particularly on SPIO-enhanced MRI, can aid in accurate diagnosis.

## Introduction

An epidermoid cyst in the intrapancreatic accessory spleen (ECIPAS) is a type of epidermoid cyst that arises within the intrapancreatic accessory spleen and generally does not require therapeutic intervention. However, ECIPAS often mimics malignant cystic neoplasm in imaging, making an accurate diagnosis challenging without postoperative pathology. As a result, surgical resection, such as distal pancreatectomy or distal splenopancreatectomy, is frequently performed [1]. When ECIPAS is correctly diagnosed, it can be observed because ECIPAS is known to lack malignant potential. Although there have been no comprehensive reports on the natural course of ECIPAS, it usually shows little change over time [2].

In this article, we present a case of a 53-year-old male patient with an ECIPAS accompanied by an increase in size over time and elevation of tumor marker serum carbohydrate antigen 19-9 (CA19-9) level, complicating clinical decision-making. Characteristic preoperative radiological imaging findings on contract-enhanced dynamic computed tomography (CT) and superparamagnetic iron oxide (SPIO)-enhanced magnetic resonance imaging (MRI), which decrease the signal intensity of splenic reticuloendothelial tissue specifically, help in the diagnosis of ECIPAS.

## Case presentation

A 53-year-old man with no medical or surgical history underwent CT and MRI to further evaluate a suspected hepatic hemangioma, which was initially detected by abdominal ultrasound. During this workup, a cystic lesion in the pancreatic tail was discovered. He was referred to our hospital for further evaluation. The patient was asymptomatic, and blood tests were normal, with a serum CA19-9 level of 21 U/mL (normal range: 0-36 U/mL).

**Figure 1:**
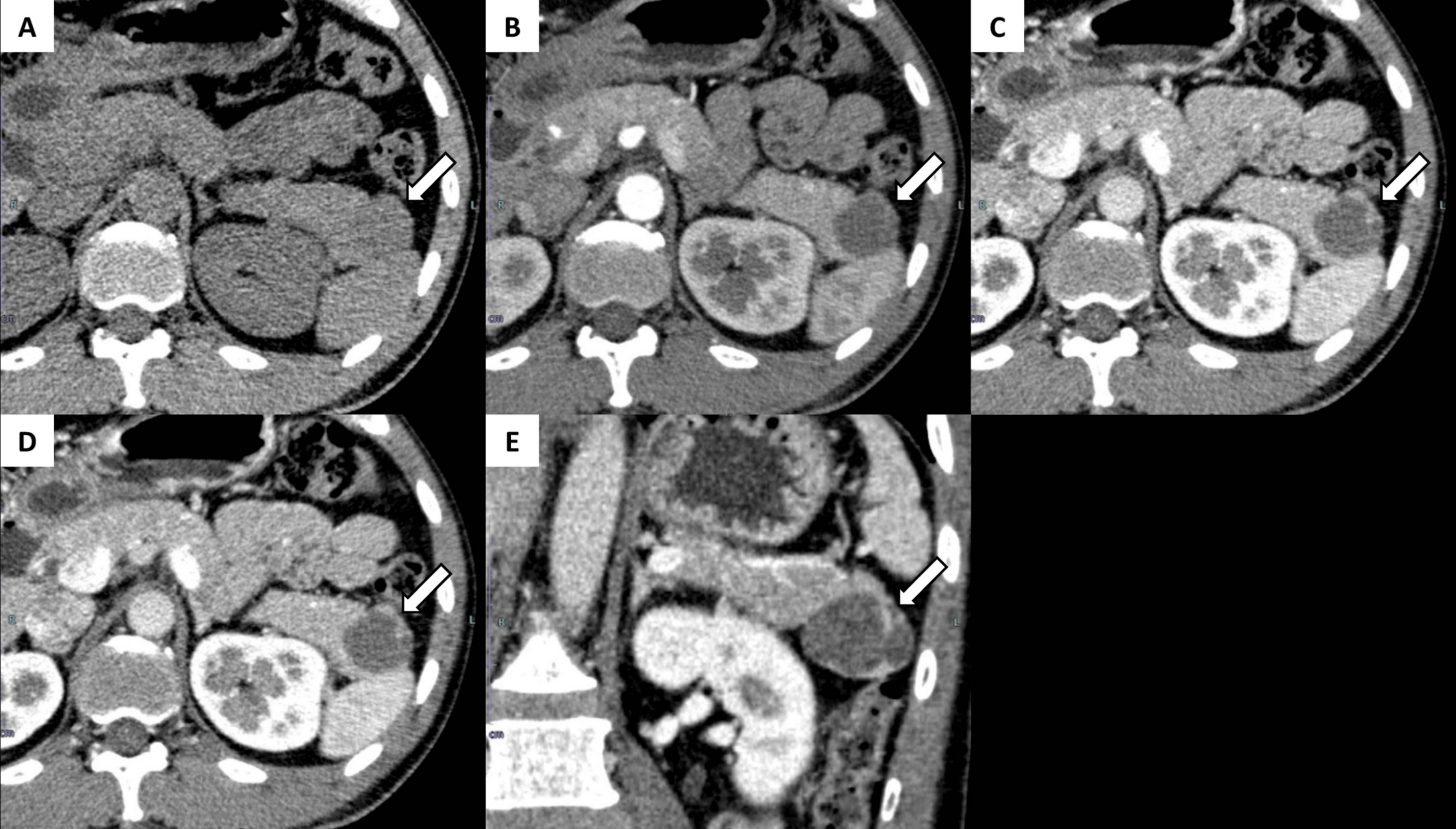
Computed tomography imaging findings (A) Unenhanced computed tomography scan reveals a hypodensity lesion in the pancreatic tail (arrow). (B-D) In the contrast-enhanced CT scan, the early arterial phase (B), late arterial phase (C), and portal phase (D) show a solid component surrounding the cyst, with a density similar to that of the spleen (arrows). (E) A coronal image of the late arterial phase shows that the pancreatic cyst measured 38 mm in diameter (arrow).

Contrast-enhanced CT revealed a predominantly cystic mass measuring 38 × 33 mm in the pancreatic tail. The lesion contained internal septations and a solid component at the periphery. The solid component showed enhancement similar to the spleen across all phases, from arterial to equilibrium (Figure 1).

**Figure 2:**
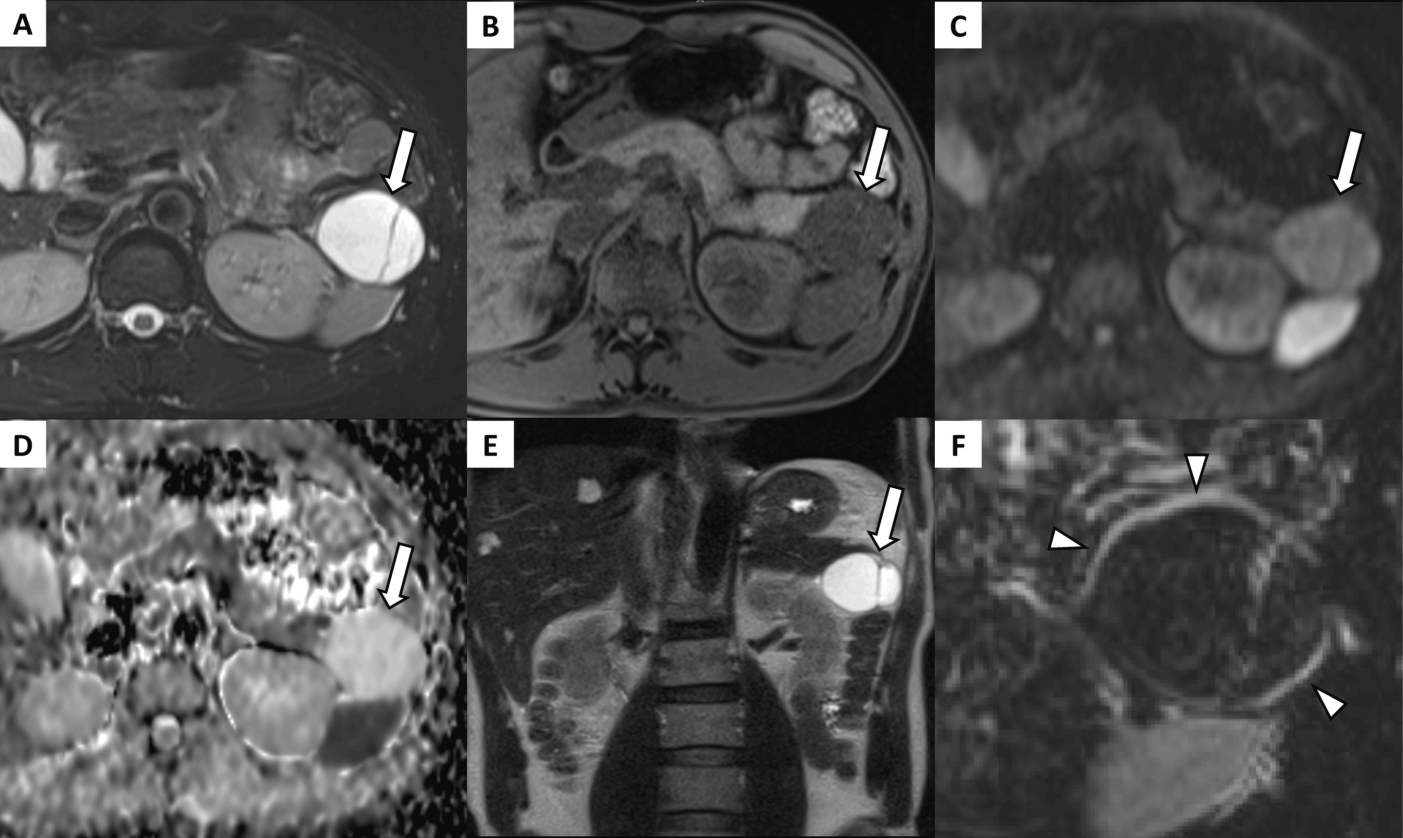
Magnetic resonance imaging findings (A) The cystic components show high intensity on fat-suppressed T2-weighted imaging with fluid-fluid levels and (B) low intensity on fat-suppressed T1-weighted imaging (arrows). (C,D) There is no diffusion restriction within the cyst (C shows diffusion-weighted imaging, and D shows the apparent diffusion coefficient map) (arrows). (E) A coronal image using half-Fourier acquisition single-shot turbo spin echo sequence (arrow). (F) A subtraction image made by subtracting superparamagnetic iron oxide (SPIO)-enhanced fat-suppressed T2-weighted imaging from pre-enhanced fat-suppressed T2-weighted imaging. The solid components show high intensity, which means a signal reduction on SPIO-enhanced fat-suppressed T2-weighted imaging, indicating SPIO uptake (arrowheads).

On MRI, the cystic component appeared hyperintense on T2-weighted imaging and hypointense on T1-weighted imaging, with no diffusion restriction. The solid component demonstrated signal reduction on SPIO-enhanced fat-suppressed T2-weighted imaging, indicating SPIO uptake (Figure 2). There was no dilation of the main pancreatic duct.

Our primary diagnosis was an ECIPAS. Other differential diagnoses included mucinous cystic neoplasm (MCN), intraductal papillary mucinous neoplasm (IPMN), serous cystic neoplasm (SCN), lymphoepithelial cyst (LEC), neuroendocrine neoplasm (NEN) with cystic degeneration, and solid pseudopapillary neoplasm (SPN). While MCN has a similar cystic morphology, it is rare in males, lowering its likelihood. IPMN was less likely due to the atypical cyst morphology and lack of communication with the main pancreatic duct. Macrocystic type SCN, LEC, NEN, and SPN can have similar imaging findings, but the presence of a solid component resembling splenic tissue with SPIO uptake made ECIPAS the most probable diagnosis. We decided to proceed with imaging follow-up.

**Figure 3:**
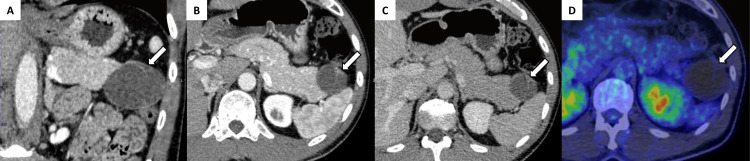
Computed tomography and 18F-FDG PET/CT imaging findings (A) Twelve months later, a coronal image of the late arterial phase reveals that the cyst had grown to 50 mm in diameter (arrow). The solid component continues to show enhancement similar to that of the spleen across all phases (arrows in B and C). B shows the late arterial phase, and C shows the portal phase. (D) 18F-FDG PET/CT showed no abnormal FDG uptake (arrows). 18F-FDG PET/CT: Fluorine-18 fluorodeoxyglucose positron emission tomography/computed tomography.

Twelve months after the initial imaging, serum CA19-9 had increased to 330 U/mL, and the mass had grown to 50 × 45 mm (Figure 3). Gastrofiberscopy and colonofiberscopy did not find any malignant lesions, and 18F-fluorodeoxyglucose positron emission tomography (18F-FDG PET)/CT showed no abnormal FDG uptake (Figure 3).

**Figure 4:**
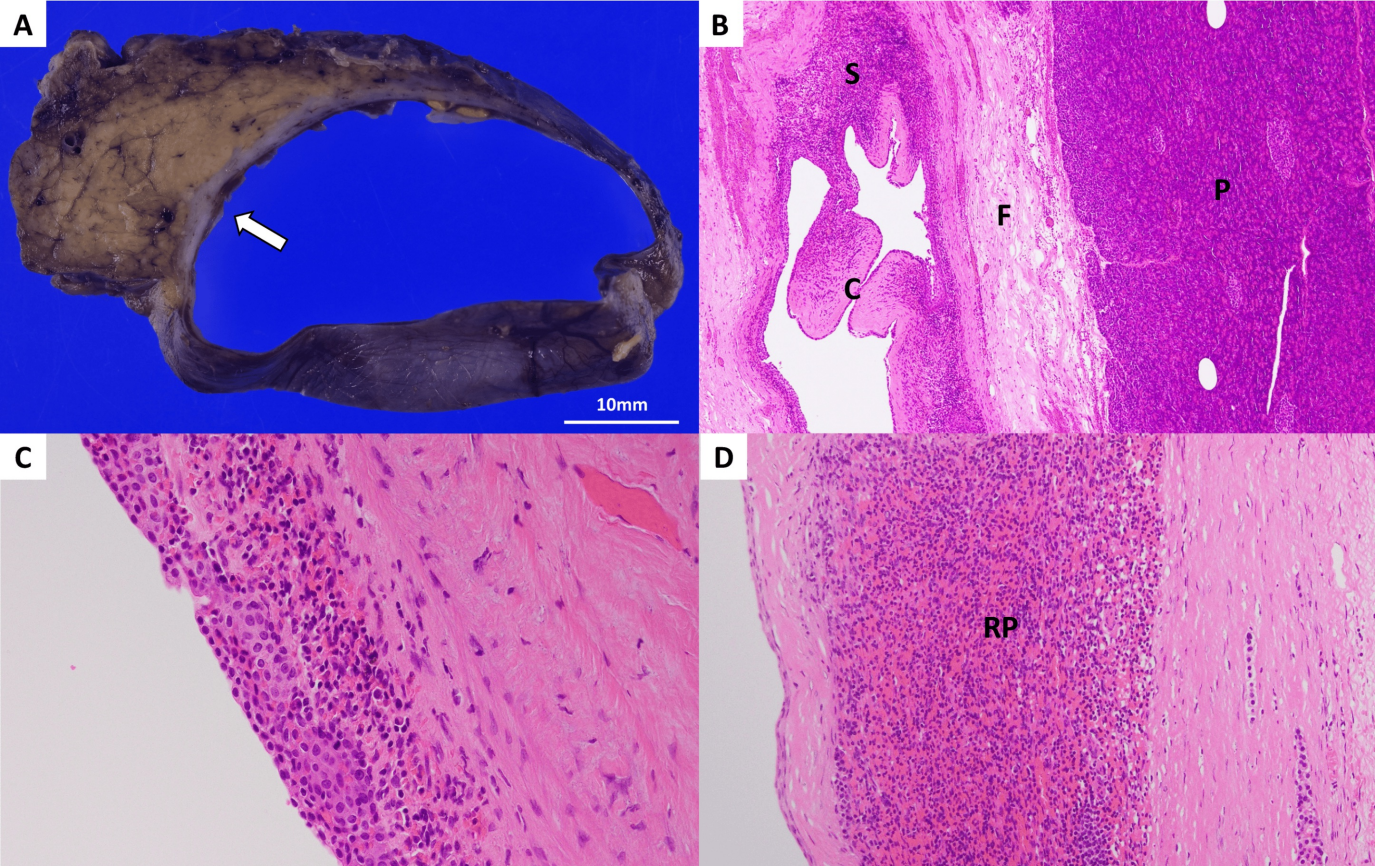
Macroscopy appearance and histopathology (hematoxylin and eosin staining) (A) Macroscopic appearance. The cyst is located at the pancreas tail in a cut section. The cyst is surrounded by a thin layer of brown tissue that looks like a normal spleen (arrow). The cyst contains keratin and serous fluid. (B) The cyst (C) is surrounded by a thin layer of splenic tissue (S) and fibrous tissue (F) adjacent to the normal pancreatic tissue (P). (C) The cyst is lined by stratified squamous epithelium. (D) The splenic tissue contains both red pulp (RP) and white pulp (not shown).

While ECIPAS remained our primary diagnosis, the size increase and elevated serum CA19-9, which are considered to be caused by the lesion, made it challenging to determine whether the lesion could continue to be observed. After the informed consent regarding the potential benefits and harm of surgery, we performed a robot-assisted spleen-preserving distal pancreatectomy. Intraoperative inspection and ultrasound confirmed that the lesion was limited to the pancreas. The excised mass was sent for histopathological examination, and the diagnosis of ECIPAS was made (Figure 4). The patient was discharged on postoperative day 4, and follow-up blood tests taken 36 days after the surgery showed normalization of serum CA19-9.

## Discussion

In this article, we present a case of ECIPAS that increased in size over time and was associated with elevated serum CA19-9 levels, mimicking pancreatic malignancy. Preoperative contrast-enhanced CT with a solid component at the periphery resembling splenic tissue and SPIO-MRI with SPIO uptake in the solid component strongly suggested ECIPAS, which was pathologically confirmed.Serum CA19-9 returned to normal levels postoperatively.

Epidermoid cysts are benign true cysts lined with stratified squamous epithelium [3]. When an epidermoid cyst develops in accessory splenic tissue within the pancreas, it is termed ECIPAS. Li et al.'s [1] systematic review of 56 ECIPAS cases found a slight female predominance (male:female ratio of 1:1.33), with all cases occurring in the pancreatic tail. However, rare cases of ECIPAS in the pancreatic head have been reported [4]. Clinically, more than half (59%) of the cases are asymptomatic and discovered incidentally, as in our case, while some present with gastrointestinal symptoms, weight loss, or abdominal masses [1]. The average size is 3.4 cm (range: 1.3-15 cm), with 59% being larger than 3 cm [1]. ECIPAS usually shows little change over time; however, an increase in size over time has been occasionally reported [2]. Our case showed growth from 38 × 33 mm to 52 × 43 mm over 14 months, mainly due to cystic component enlargement.

ECIPAS has been associated with elevated serum CA19-9 levels. While CA19-9 is recognized as a marker for malignant tumors such as pancreatic carcinoma, Li et al.'s [1] review found that 59% (20/37 cases) of ECIPAS cases had elevated serum CA19-9, although the degree of elevation was not specified. The exact mechanism of CA19-9 elevation in ECIPAS is not fully understood, but it is thought that the elevation in serum CA19-9 levels is correlated with increased intracystic pressure due to a CA19-9-rich cystic fluid produced by the epithelium lining [5]. In our case, CA19-9 levels returned to normal postoperatively, suggesting that ECIPAS was the cause of the elevation. Takagi et al. [6] also reported an ECIPAS case where CA19-9 increased to 901 U/mL and decreased to 9.9 U/mL after surgery. Therefore, it is important to note that serum CA19-9 elevation does not necessarily indicate malignancy and can occur in ECIPAS. Nonetheless, careful informed consent is needed to resect or observe pancreas cystic lesions with an increase of tumor markers in clinical practice.

On imaging, ECIPAS appears as a well-defined unilocular or multilocular cystic lesion in the pancreatic tail. Identifying the accessory spleen surrounding the cyst is crucial for diagnosis, as it should demonstrate imaging characteristics identical to the spleen across all sequences and modalities [7]. On MRI, ECIPAS typically appears hyperintense on T2-weighted images, while T1-weighted signal intensity varies from low to high depending on cyst content [1]. Diffusion-weighted imaging shows high signal intensity with low apparent diffusion coefficient (ADC) values at the lesion periphery, similar to the spleen [1,8]. SPIO-enhanced MRI is particularly useful for accurate diagnosis of ECIPAS because the solid component shows SPIO uptake similar to the spleen and appears hypointense on T2-weighted images [9]. The cystic components of ECIPAS show various ADC values depending on the internal keratin component [10]. In our case, the lesion was a predominantly cystic mass with internal septations. On contrast-enhanced CT, the solid component surrounding the cyst showed CT values similar to the spleen in all phases, and SPIO-enhanced MRI confirmed SPIO uptake in the solid component, consistent with ECIPAS. On MRI, the cystic component was hyperintense on T2-weighted images and hypointense on T1-weighted images and showed high signal intensity on diffusion-weighted images with high ADC values.

Differential diagnoses for pancreatic cystic lesions that can mimic ECIPAS include MCN, branch-duct IPMN, SCN, LEC, pancreatic NEN with cystic degeneration, and SPN. Branch-duct IPMN typically presents as dilated branch pancreatic ducts or grape-like cystic lesions that are continuous with the main pancreatic duct [11], which differs from our case. While MCN, macrocystic type SCN, LEC, cystic pancreatic NEN, and SPN can show similar imaging findings to ECIPAS, identifying a solid component with the same CT and MRI characteristics as the spleen surrounding the cyst, as in our case, can help distinguish ECIPAS from these entities. However, cystic pancreatic NEN can sometimes show enhancement similar to the spleen on contrast-enhanced CT, making differentiation challenging [12,13]. In such cases, SPIO-enhanced MRI may aid in correct diagnosis [14]. Additionally, uptake on nuclear medicine studies using somatostatin receptor-binding radionuclides such as indium-diethylenetriaminepentaacetic acid-octreotide 111 or tetraazacyclododecanetetraacetic acid-tyrosine-3-octreotide would support a diagnosis of NEN [13,15]. MCN and SPN are less likely in male patients, as they predominantly occur in middle-aged and young women, respectively [11].

## Conclusions

We report a case of ECIPAS associated with serum CA19-9 elevation and size increase, complicating clinical decision-making. An increase in size over time has been occasionally reported in ECIPAS, and serum CA19-9 elevation is observed in more than half of the cases. Correctly identifying a solid component surrounding the cyst with similar attenuation, signal intensity, and enhancement patterns as the spleen on CT and MRI, along with confirmation of SPIO uptake in the solid component, can lead to an accurate diagnosis. Given the potential benefits and risks of surgery, careful clinical decision-making is needed for pancreas cystic lesions with an increase of tumor markers in clinical practice.
